# Supplementary material for: Focused clamping of a single neuronal SNARE complex by complexin under high mechanical tension
Source: Nat Commun. 2018 Sep 7;9:3639. doi: 10.1038/s41467-018-06122-3 (PMC6128827; doi:10.1038/s41467-018-06122-3)
Supplement: Supplementary file 1 — Supplementary Information [file 41467_2018_6122_MOESM1_ESM.pdf]

Supplementary Information for:

Focused clamping of a single neuronal SNARE complex by complexin under high mechanical tension

Shon *et al.*

This PDF file includes:

Supplementary Methods

Supplementary Figures 1–8

Supplementary Tables 1–2

Supplementary References

SUPPLEMENTARY NOTES

Supplementary Note 1. Buffers for protein purification

Lysis buffers

For complexin (Cpx): 50 mM Tris-HCl (pH 8.0), 500 mM NaCl, 1 mM phenylmethylsulfonyl fluoride (PMSF), 1% Triton X-100, 2 mM dithiothreitol (DTT)

For SNAP-25 and synaptobrevin-2: The above buffer with 10% glycerol and 20 mM imidazole

For syntaxin-1A: The above buffer with 0.5% octyl glucoside and 1% sarcosine

Wash buffers

For Cpx: 50 mM Tris-HCl (pH 8.0), 500 mM NaCl, 1 mM DTT

For SNAP-25 and synaptobrevin-2: The above buffer with 10% glycerol and 20 mM imidazole

For syntaxin-1A: The above buffer with 1% sarcosine

Elution buffers

For Cpx: 50 mM Tris-HCl (pH 8.0), 150 mM NaCl

For SNAP-25 and synaptobrevin-2: The above buffer with 10% glycerol, 400 mM imidazole, and 1 mM DTT

For syntaxin-1A: The above buffer with 1% sarcosine

Supplementary Note 2. Primers for the preparation of DNA handles

Primer sequences for the biotin- and digoxigenin-modified 510-bp handles were:

Forward: 5'-/biotin or digoxigenin/ TCG CCA CCA TCA TTT CCA-3'

Reverse: 5'-/thiol/ CAT GTG GGT GAC GCG AAA-3'

Supplementary Note 3. Calculation of extension for the SNARE–DNA conjugate

The total extension we monitor in our magnetic tweezers assay, z_{bead}, is given by:

$$z_{\text{bead}}(F) = z_{\text{SNARE}}(F) + z_{\text{DNA}}(F) + z_{\text{PEG}}(F)\,, \tag{1}$$

where z_{SNARE}, z_{DNA}, and z_{PEG} are the extensions resulting from the SNARE complex, the linker DNA (two 510-bp fragments), and 5-kDa PEG, respectively. The z_{SNARE} values at different forces were estimated for the conformations below (**Supplementary Fig. 3**).

<u>Fully zippered:</u>

$$z_{\text{SNARE}}^{\text{Fully zippered}} = d_{\text{bundle}}\,, \tag{2}$$

where d_{bundle} is the width of the SNARE four-helix bundle (= 2 nm).

<u>Linker open:</u>

$$z_{\text{SNARE}}^{\text{Linker open}} = d_{\text{bundle}} + z_{\text{PP}}(11_{\text{SX}} + 12_{\text{SB}})\,, \tag{3}$$

where $z_{\text{PP}}(N)$ is the extension of unstructured polypeptide with *N* amino acids (subscripts “SX” and “SB” denote the numbers of amino acids form syntaxin-1A and synaptobrevin-2, respectively).

<u>Partially zippered:</u>

$$z_{\text{SNARE}}^{\text{Partially zippered}} = \frac{d_{\text{bundle}}}{\sin\theta} + z_{\text{PP}}(11_{\text{SX}} + N_{\text{SX}} + 12_{\text{SB}} + N_{\text{SB}})\,, \tag{4}$$

where $\theta = \text{atan}\left(d_{\text{bundle}}/\left((N_{\text{SB}} - N_{\text{SX}})h_{\text{helix}}\right)\right)$ and h_{helix} is the helical rise per amino acid (= 0.15 nm). N_{SB} and N_{SX} are the number of residues unzipped/frayed in synaptobrevin-2 and syntaxin-1A, respectively, *in addition to the linker region amino acids*, that range from 0 to 53 (28 being unzipping to the zeroth ionic layer). The half-zippered state observed in our experiments (Figs. 3a and 3g) best agreed with unzipping of synaptobrevin-2 to +2 layer ($N_{\text{SB}} = 21$) accompanied by unfolding of syntaxin-1A to +4 layer ($N_{\text{SX}} = 14$), consistent with previous tweezing experiments using optical tweezers[1]. Also, the unzipped state was

treated as another special case with $N_{\mathrm{SB}} = 53$ (complete unzipping). In this case, $N_{\mathrm{SX}} = 28$ (further fraying of syntaxin-1A to the zeroth layer) matched our data well.

Unfolded:

$$z_{\mathrm{SNARE}}^{\mathrm{Unfolded}} = z_{\mathrm{PP}}(64_{\mathrm{SX}} + 65_{\mathrm{SB}})\,. \tag{5}$$

Through Eqs. (1)–(5), z_{PP} was estimated using the worm-like chain model:

$$F = \left(\frac{k_{\mathrm{B}}T}{P_{\mathrm{PP}}}\right)\left[\frac{1}{4(1 - z_{\mathrm{PP}}/L_{\mathrm{PP}})^2} - \frac{1}{4} + \frac{z_{\mathrm{PP}}}{L_{\mathrm{PP}}}\right], \tag{6}$$

where P_{PP} is the persistence length of polypeptide (0.6 nm), L_{PP} is the contour length of the polypeptide (= 0.4 nm × [Number of amino acids in the chain]), and $k_{\mathrm{B}}T$ the thermal energy (4.11 pN·nm). Finally, z_{DNA} and z_{PEG} were calculated from similar relations:

$$F = \left(\frac{k_{\mathrm{B}}T}{P_{\mathrm{DNA}}}\right)\left[\frac{1}{4(1 - z_{\mathrm{DNA}}/L_{\mathrm{DNA}} + F/K_{\mathrm{DNA}})^2} - \frac{1}{4} + \frac{z_{\mathrm{DNA}}}{L_{\mathrm{DNA}}} - \frac{F}{K_{\mathrm{DNA}}}\right], \tag{7}$$

$$F = \left(\frac{k_{\mathrm{B}}T}{P_{\mathrm{PEG}}}\right)\left[\frac{1}{4(1 - z_{\mathrm{PEG}}/L_{\mathrm{PEG}})^2} - \frac{1}{4} + \frac{z_{\mathrm{PEG}}}{L_{\mathrm{PEG}}}\right], \tag{8}$$

where L_{DNA} (= 345 nm), P_{DNA} (= 31 nm), and K_{DNA} (= 560 pN) are contour length, persistence length, and stretch modulus of double-stranded DNA, respectively (measured independently for 1-kb double-stranded DNA), and L_{PEG} (= 36 nm) and P_{PEG} (= 0.47 nm) are contour and persistence length of PEG chain[2], respectively.

Supplementary Note 4. Analysis of force-dependent lifetimes of SNARE complexes

The distribution of lifetimes from force-jump experiments were fit to an exponential distribution to obtain the mean value, τ_{unzip} and τ_{rezip}, the reciprocals of which are the mean rates of unzipping and rezipping (k_{unzip} and k_{rezip}), respectively. The exponential dependence of rates on the external force was used to extract the location of the energy

barrier through the following set of equations[3,4]:

$$k_{\text{unzip}}(F) = k_w \exp\left[\left(-\Delta G^{\ddagger}_{\text{unzip}}(F) + F\Delta x^{\ddagger}_{\text{unzip}}\right)/k_{\text{B}}T\right], \tag{9}$$

$$k_{\text{rezip}}(F) = k_w \exp\left[\left(-\Delta G^{\ddagger}_{\text{rezip}}(F) - F\Delta x^{\ddagger}_{\text{rezip}}\right)/k_{\text{B}}T\right], \tag{10}$$

or their logarithmic representations:

$$k_{\text{B}}T \log\left(k_{\text{unzip}}/k_w\right) = -\Delta G^{\ddagger}_{\text{unzip}} + F\Delta x^{\ddagger}_{\text{unzip}}, \tag{11}$$

$$k_{\text{B}}T \log\left(k_{\text{rezip}}/k_w\right) = -\Delta G^{\ddagger}_{\text{rezip}} - F\Delta x^{\ddagger}_{\text{rezip}}, \tag{12}$$

where k_w is the diffusion-limited transition rate for protein folding[5] (= 1×10^{6} s^{-1}), $\Delta G^{\ddagger}$ and $\Delta x^{\ddagger}$ represent the height and distance of the energy barrier, respectively, and $k_{\text{B}}T$ the thermal energy (4.11 pN·nm). The values thus obtained, in turn, yielded the energy profile for the unzipping/rezipping transition (**Fig. 2h**).

Supplementary Note 5. Analysis of force-dependent equilibrium for conformational intermediates

To model the force-dependent population shift among the conformational intermediates (as plotted in **Figs. 3f** and **5d**), we considered the detailed balance between the states governed by Boltzmann relation[6]. Specifically:

$$\frac{p_{\text{LO}}(F)}{p_{\text{FZ}}(F) + p_{\text{LO}}(F)} = \left[1 + \exp\left(\left(F - F^{\text{FZ/LO}}_{1/2}\right)\Delta x^{\text{FZ/LO}}_{1/2}/k_{\text{B}}T\right)\right]^{-1}, \tag{13}$$

$$\frac{p_{\text{HZ}}(F)}{p_{\text{LO}}(F) + p_{\text{HZ}}(F)} = \left[1 + \exp\left(\left(F - F^{\text{LO/HZ}}_{1/2}\right)\Delta x^{\text{LO/HZ}}_{1/2}/k_{\text{B}}T\right)\right]^{-1}, \tag{14}$$

where p_{FZ}, p_{LO}, and p_{HZ} are the probabilities of finding a SNARE complex in the fully zippered, the linker-open, and the half-zippered state, respectively. Also, $F_{1/2}$ denotes the coexistence force between the two designated states (at which the two states are equally

populated), and $\Delta x_{1/2}$ is the distance change in the reaction coordinate at $F_{1/2}$. We obtained the $F_{1/2}$ and $\Delta x_{1/2}$ for the two equilibria using the measured values of $p(F)$, and then calculated the expected population change versus force using Eqs. (13)–(14) and the unity $p_{\mathrm{FZ}}(F) + p_{\mathrm{LO}}(F) + p_{\mathrm{HZ}}(F) = 1$.

Supplementary Note 6. Hidden Markov modelling of the high-speed bead trajectory

For kinetic analysis (**Fig. 4** and **Supplementary Fig. 7**), hidden Markov modelling (HMM) of the bead trace measured at 1.2 kHz was performed using custom MATLAB codes. For each 15-s trace, Baum-Welch algorithm was applied to compute the maximum-likelihood parameters of HMM. In this step, the locations and width of the Gaussian components given by Gaussian mixture model were used as inputs without further adjustment. Thus, only the transition matrix was optimized here. Using the obtained state probabilities and transition matrix, the most probable Viterbi path was then calculated to verify the results. The corresponding transition rates between the states were extracted to draw the detailed energy diagram.

SUPPLEMENTARY FIGURES

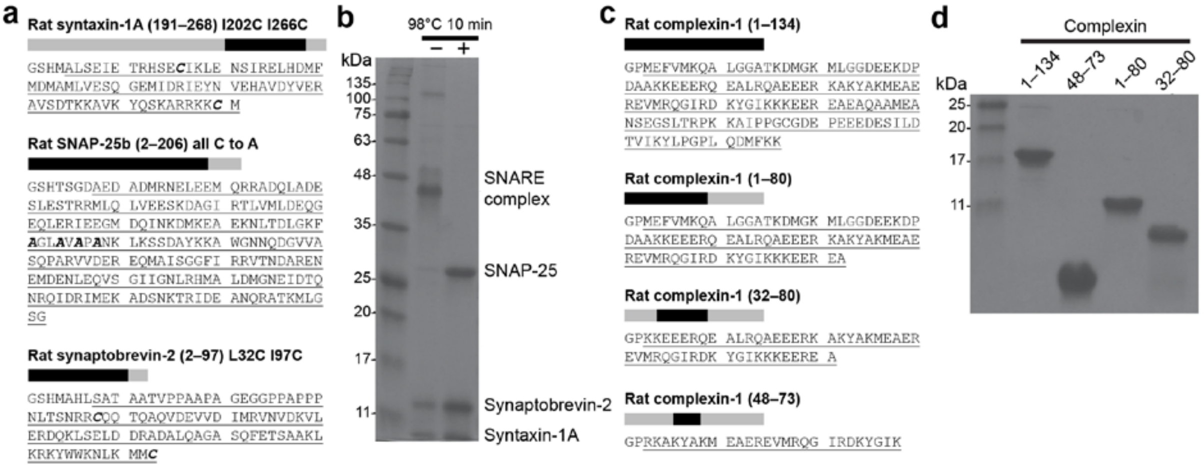

Supplementary Figure 1 Preparation of SNARE and Cpx proteins. (**a**) Amino acid sequences of the SNARE proteins used in this study. (**b**) 12% SDS-PAGE gel images of the assembled (lane "–") and the heat-disassembled (lane "+") SNARE complexes. (**c**) Amino acid sequences of the wild-type Cpx and truncation variants used in this study. (**d**) 20% SDS-PAGE gel for individual Cpx variants. In **a** and **c**, black bars mark the expressed region in the full-length wild-type sequence (grey). The underlined letters indicate the sequence that belongs to the native sequence of the respective proteins.

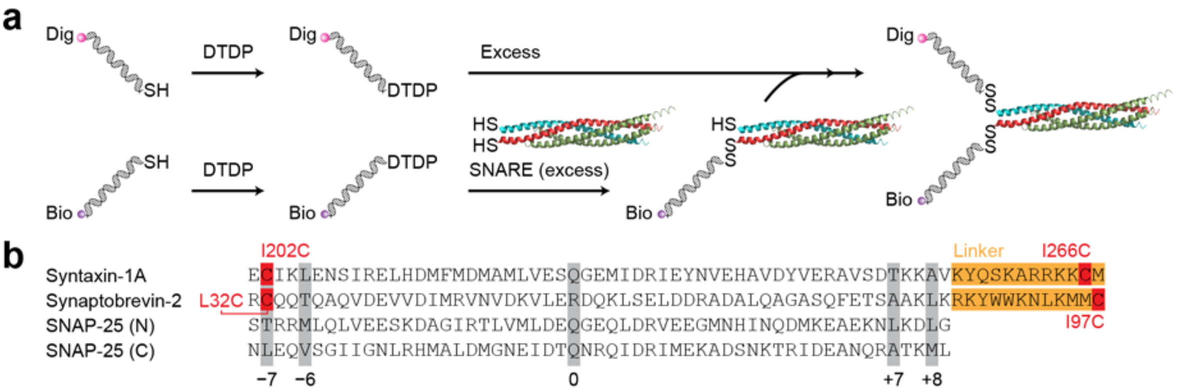

Supplementary Figure 2 Preparation of SNARE–DNA conjugate for magnetic tweezers experiments. (**a**) A scheme for the DNA handle attachment. The full details of the procedure are provided in **Methods**. (**b**) Location of artificial cysteine residues in SNARE proteins: I266 (syntaxin-1A) and I97 (synaptobrevin-2) for DNA handle attachment; I202 (syntaxin-1A) and L32 (synaptobrevin-2) for internal cross-linking of the SNARE complex.

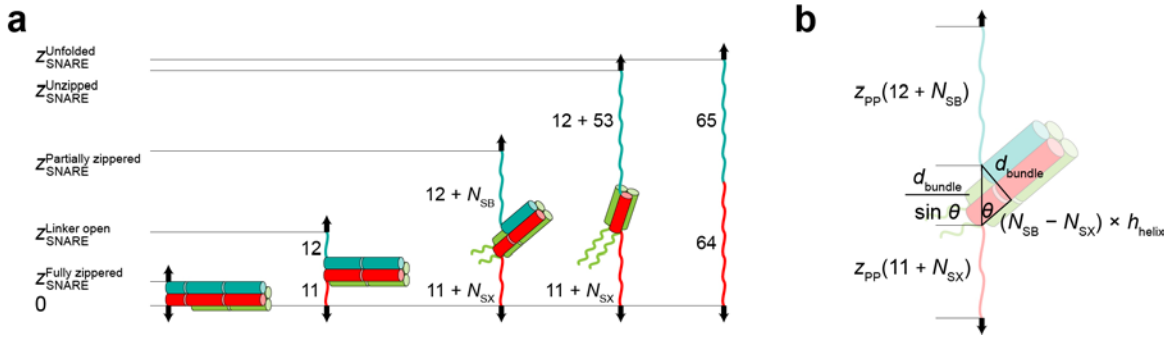

Supplementary Figure 3 Model of SNARE complex unzipping. (**a**) Intermediate conformations of a SNARE complex during unzipping. The numbers indicate the numbers of amino acids in the unfolded polypeptide segments in each conformation. (**b**) A detailed model for the extension of a partially zippered SNARE complex.

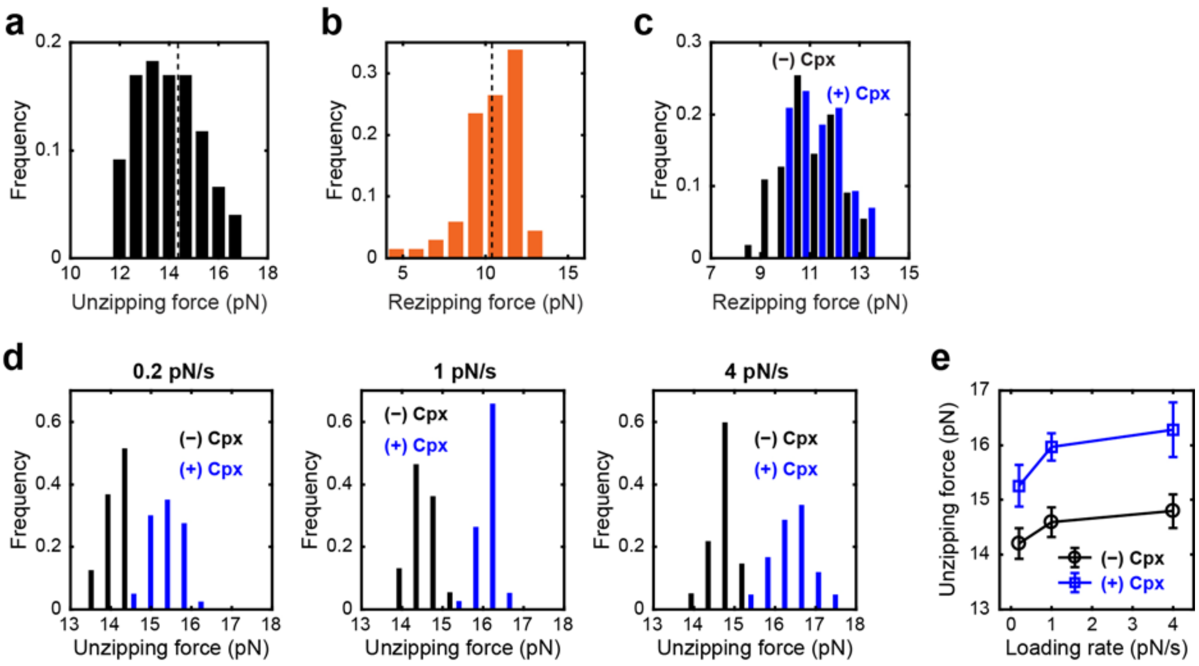

Supplementary Figure 4 Unzipping and rezipping forces of SNARE complexes and the effect of Cpx. (**a**,**b**) Distributions of unzipping (**a**; *N* = 96) and rezipping (**b**; *N* = 90) force (without Cpx) of single SNARE complexes measured with multiple magnetic beads. The global unzipping and rezipping forces (dashed lines) were 14.4 ± 1.3 and 10.4 ± 1.6 pN, respectively. The unzipping and rezipping forces in each bead were measured at least 5 times and averaged. (**c**) Distributions of rezipping force measured with a single magnetic bead in the absence (black) and presence (blue) of 5 μM Cpx. (**d**) Force loading rate dependence of unzipping force measured at 0.2, 1, and 4 pN/s. (**e**) Summary of the results in **d**, represented as means ± s.d. of the distributions in **d**.

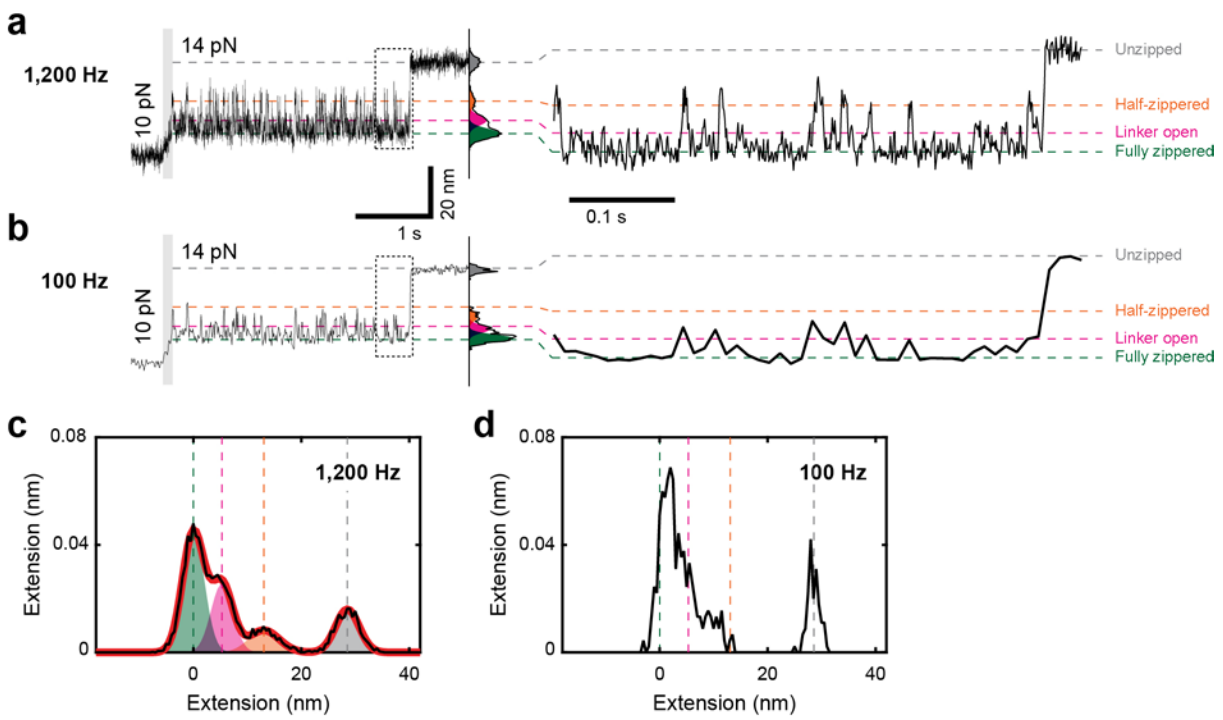

Supplementary Figure 5 Comparison of temporal resolutions for monitoring extensions of SNARE complexes. (**a**,**b**) Time traces for SNARE complex extension sampled at 1,200 Hz (**a**) and 100 Hz (**b**) in the absence of Cpx. (**c**,**d**) The corresponding distributions of extensions for the traces presented in **a** and **b**, respectively. Black lines represent the measured distributions. Dashed lines indicate the locations of the identified Gaussian components. Red line in **c** represents a fit to a Gaussian mixture model. The state locations in **d** are borrowed from **c** for comparison.

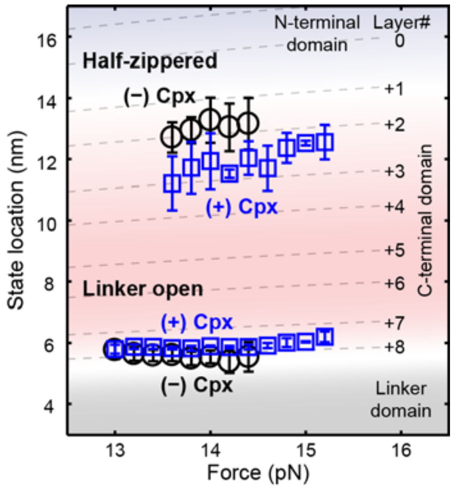

Supplementary Figure 6 Force-dependent changes in the location of linker-open (lower) and half-zippered state (upper) in the absence (black) and presence (blue) of Cpx. Dashed lines indicate the calculated distances of unzipping from the fully zippered state to the specified leucine zipper layers (**Supplementary Note 2**).

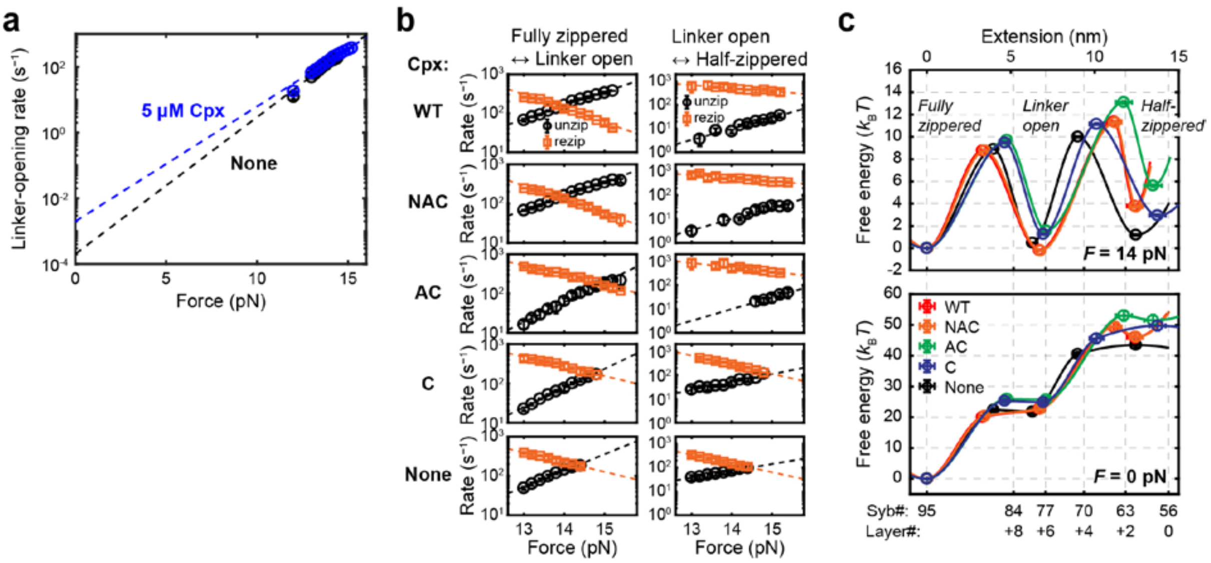

Supplementary Figure 7 Kinetic analysis of the zippered intermediates using hidden Markov modelling. (**a**) Linker-opening rates in the low-force regime. The data in **Fig. 4e** were fit to linear models and extrapolated to 0 pN. The intercepts at 0 pN were 2×10^{-4} s^{-1} without Cpx (black) and 2×10^{-4} s^{-1} with 5 μM Cpx (blue). (**b**) Force dependences of the transition rates between the zippered-state intermediates obtained from the modelled paths (such as in **Fig. 4a** in the main text) in the presence of the indicated Cpx variants. Dashed lines represent the fits to the Bell equation. (**c**) Energy diagrams for the three zippered states of SNARE complex at 14 (upper) and 0 pN (lower). The corresponding residue numbers of synaptobrevin-2 to which the SNARE complex is unzipped are indicated at the bottom.

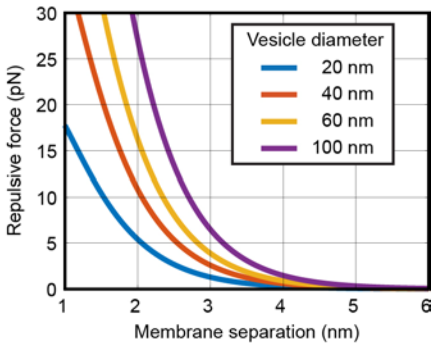

Supplementary Figure 8 Estimation of the repulsive force between a synaptic vesicle and the plasma membrane. The repulsive forces were calculated as described in a literature[7] for the specified vesicle diameters (40–60 nm being the physiological average) in the fixed-potential limit. In the estimation, the surface potentials of vesicle and plasma membrane were assumed −25 and −70 mV, respectively, and the Debye screening length of 0.67 nm was used.

SUPPLEMENTARY TABLES

Reagent	Concentration	Buffer	Incubation	Wash
**NeutrAvidin/ SNARE–DNA mix** [a]	1–10 nM [b]	PBS (pH 7.4)	10 min	200 µl PBS
**2,2′-dithiodipyridine** [c]	1 mM	Tris-HCl (pH 8.0)	5 min	200 µl PBS
**Streptavidin-coated polystyrene bead** [d]	0.2 % w/v	PBS (pH 7.4)	5 min	200 µl PBS
**Anti-digoxigenin-coated magnetic bead** [e]	0.3 mg/ml	PBS (pH 7.4) with 2.5 µM SNAP-25	10 min	200 µl PBS

Supplementary Table 1 Sample assembly procedure for magnetic tweezers experiments.

[a] SNARE–DNA conjugate was mixed with a 10-fold excess of NeutrAvidin for 30 min in advance.

[b] Concentration with regard to the biotinylated end.

[c] This step ensures the covalent coupling of the N-terminal ends of synaptobrevin-2 and syntaxin-1A.

[d] The polystyrene beads serve as fiducial markers in tracking magnetic beads.

[e] Surface coating of magnetic beads with anti-digoxigenin was carried out following manufacturer’s protocol (Thermo Fisher).

State	x (nm)	G_0 (k_BT)	$\Delta x^{\ddagger}_{\mathrm{unzip}}$ (nm)	$\Delta G^{\ddagger}_{0,\mathrm{unzip}}$ (k_BT)	$k_{0,\mathrm{unzip}}$ (10^{-4} s^{-1})	$\Delta x^{\ddagger}_{\mathrm{rezip}}$ (nm)	$\Delta G^{\ddagger}_{0,\mathrm{rezip}}$ (k_BT)	$k_{0,\mathrm{rezip}}$ (10^{5} s^{-1})
**Fully zippered**	0 (def.)<br>0 (def.)	0 (def.)<br>0 (def.)	3.9 ± 0.2<br>3.3 ± 0.1	22.3 ± 0.6<br>20.0 ± 0.4	2 ± 1<br>20 ± 8	N.A.	N.A.	N.A.
**Linker-open**	6.3 ± 0.2<br>6.7 ± 0.2	21.8 ± 0.7<br>22.6 ± 0.6	2.70 ± 0.07<br>4.4 ± 0.4	18.7 ± 0.2<br>27 ± 1	75 ± 20<br>0.03 ± 0.04	−2.3 ± 0.1<br>−3.4 ± 0.1	0.5 ± 0.3<br>−2.6 ± 0.5	6 ± 2<br>140 ± 60
**Half-zippered**	12.4 ± 0.3<br>12.4 ± 0.5	43.5 ± 0.8<br>46 ± 2	N.D.	N.D.	N.D.	−3.5 ± 0.1<br>−1.2 ± 0.2	−3.0 ± 0.4<br>3.3 ± 0.6	190 ± 80<br>0.4 ± 0.2

Supplementary Table 2 Kinetic and thermodynamic parameters for the three zippered states. Kinetic parameters in the absence (black) and presence (blue) of 5 μM WT Cpx. x and G_0: location and zero-force energy of a state relative to the fully zippered state; $\Delta x^{\ddagger}_{\mathrm{unzip}}$ and $\Delta x^{\ddagger}_{\mathrm{rezip}}$: locations of the energy barrier toward unzipping and rezipping, respectively; $\Delta G^{\ddagger}_{0,\mathrm{unzip}}$ and $\Delta G^{\ddagger}_{0,\mathrm{rezip}}$: heights of the zero-force energy barrier toward unzipping and rezipping, respectively. $k_{0,\mathrm{unzip}}$ and $k_{0,\mathrm{rezip}}$: zero-force transition rates toward unzipping and rezipping, respectively. Errors were obtained by propagating the confidence intervals for the parameter estimates in fits to the Bell equation (def.: defined; N.A.: not applicable; N.D.: not determined).

SUPPLEMENTARY REFERENCES

1. Ma, L. *et al.* Munc18-1-regulated stage-wise SNARE assembly underlying synaptic exocytosis. *Elife* **4,** e09580 (2015).
2. Dittmore, A., McIntosh, D. B., Halliday, S. & Saleh, O. A. Single-molecule elasticity measurements of the onset of excluded volume in poly(ethylene glycol). *Phys. Rev. Lett.* **107,** 148301 (2011).
3. Bell, G. I. Models for the specific adhesion of cells to cells. *Science* **200,** 618–627 (1978).
4. Tinoco, I. & Bustamante, C. The effect of force on thermodynamics and kinetics of single molecule reactions. *Biophys. Chem.* **101–102,** 513–533 (2002).
5. Yang, W. Y. & Gruebele, M. Folding at the speed limit. *Nature* **423,** 193–197 (2003).
6. Liphardt, J., Onoa, B., Smith, S. B., Tinoco, I. & Bustamante, C. Reversible unfolding of single RNA molecules by mechanical force. *Science* **292,** 733–737 (2001).
7. Bykhovskaia, M., Jagota, A., Gonzalez, A., Vasin, A. & Littleton, J. T. Interaction of the complexin accessory helix with the C-terminus of the SNARE complex: molecular-dynamics model of the fusion clamp. *Biophys. J.* **105,** 679–690 (2013).
